# Process evaluation of a district mental healthcare plan in Nepal: a mixed-methods case study

**DOI:** 10.1192/bjo.2020.60

**Published:** 2020-07-28

**Authors:** Nagendra P. Luitel, Erica Breuer, Anup Adhikari, Brandon A. Kohrt, Crick Lund, Ivan H. Komproe, Mark J. D. Jordans

**Affiliations:** Transcultural Psychosocial Organization (TPO), Nepal; Alan J Flisher Centre for Public Mental Health, Department of Psychiatry and Mental Health, University of Cape Town, South Africa; and Department of Medicine and Public Health, University of Newcastle, Australia; Transcultural Psychosocial Organization (TPO), Nepal; Department of Psychiatry, George Washington University, USA; and Transcultural Psychosocial Organization (TPO), Nepal; Alan J. Flisher Centre for Public Mental Health, Department of Psychiatry and Mental Health, University of Cape Town, South Africa; and Centre for Global Mental Health, Health Service and Population Research Department, Institute of Psychiatry, Psychology and Neuroscience, King's College London, UK; Faculty of Social and Behavioural Sciences, Utrecht University; and Research and Development Department, HealthNet TPO, Amsterdam, the Netherlands; Centre for Global Mental Health, Health Service and Population Research Department, Institute of Psychiatry, Psychology and Neuroscience, King's College London, UK; Faculty of Social and Behavioural Sciences, Department of Anthropology, University of Amsterdam, the Netherlands; and Transcultural Psychosocial Organization (TPO), Nepal

**Keywords:** Mental health, primary care, mhGAP Intervention Guide, integration, effectiveness, Nepal

## Abstract

**Background:**

The PRogramme for Improving Mental Health carE (PRIME) evaluated the process and outcomes of the implementation of a mental healthcare plan (MHCP) in Chitwan, Nepal.

**Aims:**

To describe the process of implementation, the barriers and facilitating factors, and to evaluate the process indicators of the MHCP.

**Method:**

A case study design that combined qualitative and quantitative methods based on a programme theory of change (ToC) was used and included: (a) district-, community- and health-facility profiles; (b) monthly implementation logs; (c) pre- and post-training evaluation; (d) out-patient clinical data and (e) qualitative interviews with patients and caregivers.

**Results:**

The MHCP was able to achieve most of the indicators outlined by the ToC. Of the total 32 indicators, 21 (66%) were fully achieved, 10 (31%) partially achieved and 1 (3%) were not achieved at all. The proportion of primary care patients that received mental health services increased by 1200% over the 3-year implementation period. Major barriers included frequent transfer of trained health workers, lack of confidential space for consultation, no mental health supervision in the existing system, and stigma. Involvement of Ministry of Health, procurement of new psychotropic medicines through PRIME, motivation of health workers and the development of a new supervision system were key facilitating factors.

**Conclusions:**

Effective implementation of mental health services in primary care settings require interventions to increase demand for services and to ensure there is clinical supervision for health workers, private rooms for consultations, a separate cadre of psychosocial workers and a regular supply of psychotropic medicines.

## Background

Globally, mental, neurological and substance-use disorders are among the leading causes of disability, contributing to 10.4% global disability-adjusted life-years.^1^ The burden of these disorders has consistently risen in the context of major demographic and socio-political transitions.^2^ Although there is an increasing evidence base for mental health interventions there is a significant gap between the number of people in need of mental healthcare and those actually receiving treatment. A recent World Health Organization (WHO) World Mental Health Survey reported that 86.3% of people with anxiety, mood or substance disorders in low- and middle-income countries (LMICs) have not received any treatment in the 12 months preceding the survey.^3^ Among those who receive treatment, only a few get adequate treatment.^4^ A recent study conducted in 21 countries reported that 1 out of 27 people living with depressive disorder in LMICs receives minimally adequate treatment.^5^

In Nepal, there is no nationally representative data on prevalence of mental disorders, however, studies conducted with specific populations or populations affected by conflict or humanitarian emergency reported high prevalence rates of mental disorders (i.e. depression, 14.0–80%; anxiety, 22.9–81.0%; and alcohol use disorder (AUD) 1.5–25%)^6,7^ and access to mental health services is extremely low (i.e. only 8.1% people with depression and 5.1% people with AUD received treatment from any providers in the past 12 months).^8^

## Mental Health Gap Action Programme

Evidence shows that mental health services can be delivered effectively by trained non-specialists healthcare providers through community-based programmes.^9,10^ The integration of mental health services in community and primary healthcare (PHC) settings has also been advocated as a strategy to reduce the treatment gap, particularly in LMICs, where mental health specialists are limited. The WHO launched the Mental Health Gap Action Programme (mhGAP) in 2008 and the mhGAP Intervention Guide in 2010,^11^ with the aim of providing evidence-based clinical guidance to PHC workers for detection, diagnosis and treatment of mental disorders in primary care. As part of PRogramme for Improving Mental Health CarE (PRIME),^12^ we developed a mhGAP-based district mental healthcare plan (MHCP) by involving a wide range of stakeholders. The MHCP comprised intervention packages to be implemented at community, health facilities and health service organisation levels.^13^

The community-level intervention packages included a community sensitisation programme, case detection in the community by using the Community Informant Detection Tool (CIDT),^14^ treatment adherence support through home-based care and community counselling. The health-facility-level packages included training and supervision of healthcare providers to detect, diagnose and treat mental disorders based on the WHO mhGAP Intervention Guide.^11^ The health-organisation-level packages included human resource mobilisation, procurement and supply of psychotropic medicines and referrals for specialised care. Details of the MHCP components are published elsewhere.^13^

## Aims

Our prior studies demonstrated a significant impact of the district MHCP on treatment coverage, detection of mental disorders in primary care and initiation of minimally adequate treatment after diagnosis and small-to-moderate effect sizes on individual-level treatment outcomes after introduction of the district MHCP.^15^ These research findings and the available literature on mental healthcare describe what ‘works and what did not work’, but there is a lack of knowledge on how a particular intervention was implemented taking into account (possible) barriers and facilitating factors. This paper aims to describe the implementation process, particularly the barriers and facilitators of the district MHCP, and evaluate the measures related to the implementation process defined by the theory of change (ToC).

## Method

### Setting

Nepal, one of the poorest countries in South Asia, has a total population of approximately 26.4 million and life expectancy at birth of 69.1 years. Nepal's gross national income per capita at purchasing power parity was $2500 in 2017, ranking 193 out of 226 countries. The district MHCP was implemented in Chitwan, a district in southern Nepal. The total population of Chitwan is 579 984 with a literacy rate of 77%, which is higher than the national average of 57%. Although a variety of caste/ethnic groups reside in Chitwan, Brahmin (28.6%), Chhetri (11.4%) and Tharu (10.9%) are the dominant groups. Chitwan district was chosen as mental health specialists are available in the district hospital and private hospitals. The MHCP was implemented in three overlapping phases: pilot testing (2 health facilities), implementation and evaluation (10 health facilities) and scaling up (34 health facilities).

### Study design

We used the ToC approach^16^ to develop the district MHCP and an evaluation framework.^17^ A ToC describes how a programme or an intervention brings desired long-term outcomes through a logical sequence of short-term and intermediate outcomes.^18^ In recent years, ToC has increasingly been used for designing and refining interventions, and as a framework for evaluation.^19^ We conducted four ToC workshops with national and district-level stakeholders including mental health specialists and primary care workers in order to develop the MHCP, and the related evaluation framework.^20^ In the first two ToC workshops (district-level stakeholders, *n* = 14 and policymakers, *n* = 10), we determined short-term and intermediate outcomes, interventions and assumptions to achieve the overall impact of the district plan. Stakeholders in the last two ToC workshops (district-level stakeholders, *n* = 11 and policymakers, *n* = 8) defined indicators to measure each of the MHCP intermediate outcomes. These indicators were used to assess whether key stages in the causal pathways of MHCP are achieved. Details of the ToC can be found in Breuer et al.^20^

The MHCP was evaluated using multiple methods, including pre- and post-community- and health-facility-based surveys, cohort studies and process evaluations of implementation of the care plans.^17^ Pre- and post-cross-sectional community surveys were conducted to assess changes in treatment contact coverage, pre- and post-facility-based surveys were conducted to measure changes in detection and initiation of minimally adequate treatment by trained PHC workers. The cohort studies were done to assess the impact of mental health services on patients’ clinical, social and economic outcomes. The results of these studies are reported elsewhere.^15^ The implementation process, particularly the barriers and facilitators of the MHCP, and indicators related to the implementation process, was evaluated using a case study method.^17^

The case study evaluated the input and process indicators defined by the ToC,^21^ which are not otherwise captured by the community, facility detection surveys and cohort studies described in the paragraph above.^15^ The case study assessed; (a) the social, political, economic and cultural context that may affect the implementation of the MHCP; (b) the availability of physical, human and financial resources required for the implementation of the MHCP; (c) the implementation process of the MHCP including reach and coverage of the services; (d) the training and supervision of the service providers implementing the MHCP; (e) the perspectives of service providers, patients and caregivers on the acceptability and feasibility of the services; and (f) the barriers and facilitating factors for the implementation of the care plan. A range of qualitative and quantitative methods were used in data collection including; (a) district and community profiles, (b) health facility profiles, (c) monthly implementation logs, (d) training and supervision evaluation, and (e) in-depth qualitative interviews. Details of the different methods are presented in Table 1.

**Table 1:** Summary of data-collection methods for process evaluation

Methods	Timing	Data	Sources of data
District and community profiles	Annually and quarterly (2013–2016)	Sociopolitical and contextual challenges for implementation of mental healthcare plan Financial and human resources allocated to mental health in the district Availability of policies, guidelines and treatment protocols Types of mental health services available in the community setting	Quarterly district and community profiles Reports produced by district public health office Observation Interviews with the senior officers in the district public health office
Health facility profiles	Quarterly (2013–2016)	Physical facilities (such as separate and confidential room for consultation and providing psychosocial support) and facility operating time Number of trained health workers Available mental health services Availability of psychotropic medicines Availability of treatment protocol and guidelines in the health facilities Number of patients attending primary healthcare facilities	Semi-structured interviews with senior facility manager or in-charge personnel Out-patients’ register and facility reports Observation (physical facilities and availability of checklists, guidelines, medicine supply)
Monthly implementation logs	Monthly (2014–2016)	Number of health workers trained Number of supervision sessions Number of community sensitisation programmes conducted and number of participants Number of people initiating evidence-based mental healthcare	Monthly implementation logs
Training evaluation	Before and after the training (2014)	World Health Organization's pre- and post-test training evaluation questionnaire – to assess knowledge and attitudes^11^ Mental illness: clinician's Attitude^22^ to assess health workers’ attitude towards learning about mental healthcare and whether providing mental health services is comparable in acceptability and worth as physical healthcare ENhancing Assessment of Common Therapeutic factors – to assess clinical competence of primary healthcare workers	Pre- and post-training evaluation
Qualitative interviews	Two years after implementation of the MHCP (2016)	Experiences of service providers in delivery of specific component of mental health services (for example psychological interventions) Patients satisfaction with the services Barriers with respect to treatment engagement, adherence and retention in care Impact of the services in their day-to-day activities	Semi-structured interviews with service providers, patients and caregivers Semi-structured interviews were conducted with 47 purposively selected trained primary healthcare workers (35 prescribers and 12 non-prescribers) and 8 female community health volunteers^a^
Theory of change (ToC)	2013–2014	ToCs maps to develop a structural logical map of preconditions (or preliminary outcomes), assumptions and interventions leading to an ultimate outcome as well as outcome indicators^23^	ToC workshops with policymakers and primary healthcare workers

a. Permitting data saturation.

### Data analysis

The data were analysed using the following methods. Descriptive statistics such as percentages and proportions were used to analyse the quantitative process and input data from the facility, community and district profiles and the implementation logs. For the training evaluation data, we compared calculated percentages of correct response for knowledge and mean scores for attitude and efficacy, and individual items scores were calculated for ENhancing Assessment of Common Therapeutic factors.^24^ Pearson correlation and paired *t*-test were used to test changes between pre- and post-training evaluation. The qualitative interviews were audio-recorded first and transcribed in the original language (Nepali) by the interviewers. The transcriptions were then translated into English by professional translators. Qualitative data were analysed thematic content analysis methods using NVivo.

### Ethics

This study received ethical approval from the Nepal Health Research Council (Ref. No. 162/2015), the national ethical body of the government of Nepal; the ethical review board of WHO Geneva, and the University of Cape Town (HREC Ref: 412/2011). Written and oral information was provided to each of the study participants about the objectives and process of the study. Consent was also obtained from facility managers to use health-facility-level data. Participants provided written consent to confirm their participation. Only those people who voluntarily agreed to participate were included in the study. As interviews were planned to be audio-recorded, this was explicitly included in the informed consent procedure.

## Results

### What was achieved?

#### Mental health case-load in PHC

**Fig. 1:**
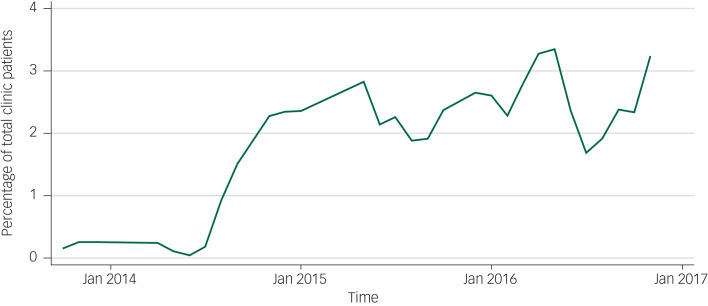
Percentage of people receiving mental health services as a percentage of all people attending primary care services over time.

Figure 1 presents the proportion of the total patients attending primary care that received mental healthcare. The proportion was very low (0.15%) before implementation of the MHCP and increased to 3.24% 3 years after implementation. The trained health workers also reported in the qualitative interviews that before introducing the MHCP, no one was aware about mental illness and its treatment process. However, after conducting awareness programmes with a range of key stakeholders in the community, people slowly started coming to health facilities for treatment.

‘In the older days, mental health was not seen as a problem, people were thinking that it doesn't need any sort of treatment. But later, the TPO [Transcultural Psychosocial Organisation] visited different healing places like traditional healers, and community leaders, volunteers, and then people started to learn about it.’ (PHC worker-13)

**Fig. 2:**
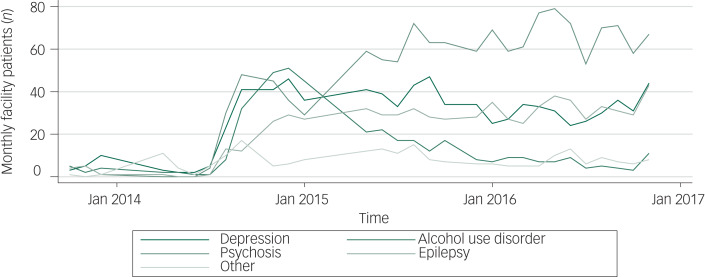
Number of people receiving treatment from primary healthcare over time by disorder.

Figure 2 shows the longitudinal trend of service utilisation for different disorders. Overall, the number of patients receiving mental healthcare increased substantially after the mhGAP-based training was initiated in early 2014. The number of patients receiving treatment for psychosis remained highest in most of the quarters except in January 2015. The number of patients with AUD decreased dramatically after 2015. This trend is also supported by the experience of trained health workers. Health workers reported that initially many people with AUD visited health facilities for treatment, and many of them stopped drinking after the treatment. However, after a few months, many people who were treated successfully started drinking again and did not come for follow-up. Therefore, health workers stopped initiating diagnosis and treatment of AUD when someone comes without family members and does not show commitment to quit alcohol forever. This narrative could explain why only a few health workers initiated treatment for patients with AUD after 2015.

‘In my experience, among the regular cases in this facility, the hardest to manage are the AUD cases because many patients go home and start drinking again, they relapse often. Then, we feel bad as service providers because the service users start again. The service users will not come back for treatment because they will feel guilty of relapsing after having regular treatment and they will be scared to face eyes with us, they will feel guilty and ashamed.’ (PHC worker-11)

#### Continued care

Table 2 presents details of the follow-up visits of patients receiving mental health services from PHC. On average, patients visited health facilities 7.1 times for follow-up, and there was a large variation in the number of follow-up visits by disorder. For example, people with epilepsy made an average of 14 visits, whereas it was only 5.0 visits for depression, 12.2 visits for psychosis and 3.0 visits for AUD. To motivate patients to come for follow-up, female community health volunteers (FCHVs) were trained in home-based care, where they discussed with both the patients and the family members about the importance of follow-up care.^13^

**Table 2:** Patients follow-up visits by disorders over 2.5 years (July 2014 to January 2017)

Number of visits	Depression, *n* (%)	Psychosis, *n* (%)	Alcohol use disorder, *n* (%)	Epilepsy, *n* (%)	Comorbidity, *n* (%)	All, *n* (%)
1 time	148 (39.1)	56 (27.2)	109 (37.3)	30 (27.0)	15 (22.4)	358 (33.9)
2–4 times	120 (31.7)	39 (18.9)	120 (41.1)	19 (17.1)	18 (26.9)	316 (30.0)
5–7 times	37 (9.8)	14 (6.8)	44 (15.1)	10 (9.0)	5 (7.5)	110 (10.4)
>7 times	74 (19.5)	97 (47.1)	19 (6.5)	52 (46.8)	29 (43.3)	271 (25.7)
Mean (range)	5.0 (1–47)	12.2 (1–49)	3.0 (1–13)	14.0 (1–70)	11.3 (1–47)	7.1 (1–70)
Total	379 (100)	206 (100)	292 (100)	111 (100)	67 (100)	1055 (100)

In the qualitative interviews, both patients and caregivers highlighted that the availability of mental health services (both psychological and pharmacological) free of charge was the most important facilitating factor for follow-up care in their community. One of the caregivers expressed that he was ‘extremely happy’ that he was able to get such quality service in his own place.

#### Progress towards other indicators

Table 3 presents the indicators for other MHCP components, intended outcome indicators and supporting evidence. It shows that the programme was successful in achieving most of the indicators defined by the ToCs. Out of six health-organisation-level indicators, four indicators (67%) were fully achieved. Six new psychotropic medicines that were used in PRIME are now included in the Ministry of Health (MoH) essential medication list, and the MoH has allocated a separate budget for scaling of mental health services. Out of 15 health-facility-level indicators, 9 indicators (60%) were fully achieved, 5 (33%) partially achieved and 1 (7%) was not achieved at all. All health workers (both prescriber and non-prescriber) from the implementation area were trained and supervised regularly. Out of six psychotropic medicines, five medicines were available most of the time in all health facilities. At the community level, we were able to fully achieve 8 (73%) out of 11 indicators. We trained and mobilised all FCHVs (*n* = 103) and 14 psychosocial counsellors. Psychosocial counsellors provided services to all patients referred by trained health workers and FCHVs. FCHVs made more than 1800 home visits. However, home visits did not achieve the intended outcome relating to drop-out rate (Table 3).

**Table 3:** Achievements against theory of change (ToC) indicators

MHCP component, ToC indicators	Indicators achieved	Supporting evidence
*Health organisation level*		
Engagement and advocacy		
Mental health is integrated in the district health plan	Fully achieved^a^	MoH has allocated separate budget for scaling up of mental health services
Mental health programme coordinator in post	Fully achieved	MoH appointed a focal person to coordinate PRIME activities in the beginning of the project DPHO appointed a focal person to coordinate MH activities in the field Mental health focal unit has been established under NCD by MoH^a^
Policy for provision of psychotropic medication	Fully achieved^a^	6 new psychotropic medicines are included in the essential list
DPHO has allocated required budget for psychotropic medicine	Fully achieved	Municipalities/village municipalities have allocated budget for psychotropic medicines
Referral for specialist's consultation		
Referral system established with the district hospital	Partly achieved	Referral system from PHCs to psychiatrist department at district hospital was established
Cases referred to psychiatrist from the PHC facilities	Partly achieved	24 people referred by PHC workers for specialised care
*Health facility level*		
Service providers awareness and anti-stigma		
Training conducted	Fully achieved	4 training courses conducted (2 for prescribers and 2 for non-prescribers)
Health workers trained	Fully achieved	35 prescribers, and 41 non-prescribers were trained (all health workers from 10 primary healthcare facilities)
Improvement knowledge and attitude of primary care workers	Partly achieved	Knowledge and attitude changed significantly after the training (see Table 4)
Screening and assessment		
Adequate numbers of human are available at the health facility levels	Partly achieved	43 prescribers and 41 non-prescribers. No psychosocial workers in the existing health system so hired counsellors externally
Staff gained knowledge and skills to diagnose and treat mental health problems	Partly achieved	Knowledge and attitude changed significantly after the training (see Table 4) Correct diagnosis and initiation of treatment changed significantly^25^
Physical/confidential space is available	Not achieved	No confidential place in most of health facilities
Protocols and guidelines are in place	Fully achieved	mhGAP Intervention Guide translated and adapted for Nepal Standard treatment protocol Trainers and facilitators manual (for both prescribers and non-prescribers) HAP and CAP manuals OPD registers/OPD card
Increased number and proportion of people identified/diagnosed	Full achieved	0.15% to 3.24% (see Fig. 1)
Basic psychosocial support		
Non-prescribers trained on basic psychosocial support	Fully achieved	All 41 non-prescriber were trained
People initiating treatment in primary care	Fully achieved	1122 (379 depression; 292 alcohol use disorder, 206 psychosis, 111 epilepsy and 134 others)
Increased number of people receiving evidence-based treatment	Fully achieved	See Fig. 2
Psychotropic treatment		
Medications were available at all clinics 95% of time	Partially achieved	Out of 6 medicines, 5 medicines were always available in all health facilities
Stock-outs in past 30 days for essential psychotropic medications outlined in the MHCP	Partially achieved	Trihexyphenidyl was not available at 4 out of the 10 facilities
Continue care		
Functioning supervision and quality control system is in place	Fully achieved	1 individual supervision 8 monthly/quarterly supervisions 2 three-days refresher training
Treatment outcomes		
Improved health, social and economic outcomes for people living with priority mental disorders	Fully achieved	Changes in treatment outcomes – small-to-moderate effect sizes (9.7 – points reduction (*d* = 0.34) in AUD symptoms, 6.4-points reduction (0.43) in psychosis symptoms and 7.2-points reduction (*d* = 0.58) in depression symptoms) at 12-months post-treatment^25^
*Community level*		
Mass community sensitisation		
Community sensitisation programme conducted	Fully achieved	139 community sensitisation programmes conducted
People oriented on mental health	Fully achieved	5628 key community members oriented on mental health
Improved mental health literacy and decreased stigma	Partly achieved	Mental health literacy increased from 22.2% to 30.4% among general community members Stigma associated with mental health decreased but the change was not significant^6^
Improvement in treatment coverage	Partially achieved	Depression, 0% to 12.2% Alcohol use disorder, 0% to 7.5% Psychosis, 3.2% to 53.4%, Epilepsy, 1.3% to 13.0%
Community detection		
FCHV trained on CIDT	Fully achieved	All 103 FCHVs were trained on CIDT
People referred through CIDT	Fully achieved	685 people were referred to health facilities through CIDT
People visited health facilities because of CIDT	Fully achieved	67%^26^
Advanced psychosocial counselling		
Psychosocial counsellors trained	Fully achieved	14
People received service from psychosocial counselling	Fully achieved	152 (see Jordans et al 2019 for details)^25^
Home-based care (HBC)		
FCHVs trained on HBC	Fully achieved	All 103 FCHVs trained on HBC
Home visits by FCHVs	Partially achieved	FCHVs made 1803 visits

MHCP, mental healthcare plan; MoH, Ministry of Health; PRIME, PRogramme for Improving Mental Health carE; DPHO, district public health office; NCD, non-communicable disease; PHC, primary healthcare; mhGAP, Mental Health Gap Action Programme; HAP, healthy activity programme; CAP, counselling for alcohol problems; OPD, out-patient department; FCHV, female community health volunteers; CIDT, Community Informant Detection Tool.

a. Achieved by the end of the project period.

#### Evaluation of training

Table 4 presents pre- and post-training evaluation results among prescriber-level health workers. The results demonstrated significant improvement in mental-health-related knowledge, attitudes and clinical competencies after the 10 days of mhGAP-based training. However, at the post-training evaluation, only 71% mean knowledge and 81% mean competency was achieved, suggesting that there remains a need for improvement in knowledge and competency.

**Table 4:** Training assessment outcomes, measured on the first and last day of training for prescriber health workers (*n* = 35)

Domain	Tool	Items, *n*	Scoring	Pre-training, mean	Pre-training, s.e.	Post-training, mean	Post-training, s.e.	Paired *t*-test	*P*
Knowledge	mhGAP Knowledge Assessment adapted for PRIME	33	True false, and multiple-choice questions	59.13%	1.55	70.56%	2.31	3.89	<0.001
Attitudes	mhGAP Attitudes adapted for PRIME	25	Likert scale, 1–4	2.28	0.04	2.10	0.03	−3.86	<0.001
Attitudes	Mental Illness: Clinicians Attitudes	16	Likert scale, 1–6	68.13	1.49	73.77	1.49	3.05	0.004
Clinical competency	ENhancing Assessment of Common Therapeutic Factors	18	Competency levels, 1–3	58.66%	2.77	81.21%	2.51	7.80	<0.001
Self-efficacy	mhGAP self-efficacy adapted for PRIME	34	Likert scale, 1–5	2.51	0.12	4.61	0.09	16.46	<0.001

mhGAP, Mental Health Gap Action Programme; PRIME, Programme for Improving Mental Health Care.

The improvements in knowledge, attitudes and competencies among health workers have also been supported by the experience of the patients and caregivers. Many patients reported that the health workers were knowledgeable and skilful. The caregivers held the perception that if the health workers were not competent, there would not have been improvement or positive changes in the patients’ condition. The patients and their caregivers perceived the health workers to be competent because of the positive change in the patients’ health.

### What was implemented?

Table 5 presents the overview of the district MHCP, implementation processes for each of the intervention packages, the role of PRIME, and the barriers and facilitating factors for successful implementation. The MHCP was implemented within the existing community and PHC system. Medical officers, health assistants and auxiliary health workers were responsible for detection and management of mental disorders by using the mhGAP Intervention Guide in PHC facilities. Staff nurses and auxiliary nurse midwives provided psychosocial support in the health facilities. FCHVs and community counsellors implemented the treatment packages in the community (see Table 5).

**Table 5:** Overview of mental healthcare plans, delivery process, prime role, barriers and facilitators

MHCP component, delivery process	PRIME support process	Barriers	Facilitating factors
Health organisation level			
Engagement and advocacy			
Mental health experts, policymakers, PHC workers and patients were involved in development of the MHCP	Organising workshops and consultative meeting with concerned stakeholders, and logistics management	No mental health focal person/unit in the MoH Mental health was lower in priority in the government system No separate budget allocated for mental health	Appointment of a senior-level MoH officer as a focal person to coordinate PRIME activities Involvement of MoH in the implementation process Implementation of the evidence-based intervention packages
Referral for specialist consultation			
Trained health workers referred difficult cases or those requiring specialists care to psychiatric ward in the district hospital	Encourage health workers to refer difficult cases to the specialists Establish formal collaboration between PRIME and district hospital	Busy schedule of psychiatrists because of high client flow Medicines provided by the psychiatrist did not match with the medicines available in the PHC facilities	Availability of specialist mental health services in the district hospital Supportive role of psychiatrists from the district hospital
Health facility level			
Service providers awareness raising and stigma reduction			
Both psychiatrist and psychologists delivered the training Sufficient time was allocated to discuss stigma and basic information about mental illness	Conducting of training programmes Logistics management for the training	Logistics for the training (daily allowance etc.) Frequent transfer of trained health workers Mental health stigma among PHC workers	Motivation of health workers to learn about mental health Supportive role from MoH and DPHO
Screening and assessment			
Health workers were trained on WHO mhGAP intervention guides Sufficient time was allocated for role-play and practice sessions	Conduction and logistics management for the training Protocol and guidelines development Clinical supervision	Lack of confidential place for assessment and consultation in the health facilities Lack of sufficient time provided by health workers for assessment because of heavy client flow Frequent transfer of trained health workers	Easy and user-friendly flow chart in the mhGAP Intervention Guide Motivation of health workers to learn about mental health Supportive role of psychiatrists to provide phone supervision to the trained health workers
Basic psychosocial support			
Psychologists/clinical supervisors conducted 4 days training on communication skills and basic psychosocial support to both prescribers and non-prescribers Sufficient role-plays and practice session were conducted	Conducting and logistics management for the training Protocol and guidelines development Clinical supervision	Prescriber-level health workers did not have much time to provide psychosocial support No separate rooms/space for providing basic psychosocial support	Motivation of health workers to learn about mental health Health workers considered psychosocial component as an important element in mental healthcare
Focus psychosocial support			
Non-prescribers received 5-days training on healthy activity programme and counselling for alcohol problems Received monthly/quarterly supervision from psychosocial counsellors	Conducting of training and logistics management Protocol and guidelines development Clinical supervision	No separate rooms for psychological intervention in the health facilities Lack of coordination between prescribers and non-prescribers	Motivation of health workers to learn about mental health Health workers considered psychosocial component as an important element in mental healthcare
Psychotropic treatment			
Initially PRIME procured and distributed of the medicines After necessary revision in the essential drugs list, medicines were distributed by DPHO through the existing system	Financial support for drugs Distribution and record-keeping Monitored stock register and buffer stock	Lengthy procurement process and distribution system Frequent transfer of senior-level officers in the MoH and DPHO	Supportive role of DPHO memorandum of understanding with MoH Primary Health Care Revitalization Division facilitated the process
Community level			
Mass community sensitisation			
FCHVs and community counsellors conducted 2–3 h sensitisation programmes in the community Posters, leaflets and brochures were distributed	Logistics management Supervision of FCHVs	Huge stigma associated with mental illness Low mental health literacy Myths and misconception on mental illness Cultural beliefs and practices	Supportive role of community members Motivation of community members to learn about mental health
Community detection			
Two days training for FCHVs on CIDT Monthly/quarterly supervision	Training and supervision	Difficult to use CIDT by illiterate FCHVs Incentives for FCHVs	Motivation of FCHVs FCHVs as a part of the existing healthcare system Knowledge of FCHVs about the community
Advanced psychosocial counselling			
PHC workers referred cases requiring psychological intervention to community counsellors Community counsellors visited respective health facilities or patient's house for delivering services	Training and supervision of community counsellors Salary and other logistics management for community counsellors	No separate rooms for counselling in most of the health facilities Stigma associated with mental illness to provide services in the community	Locally hired counsellors Support from trained PHC workers
Home-based care (HBC)			
FCHVs received 2-day training on HBC Supervision of FCHVs by community counsellors	Training and supervision of FCHVs Logistics management for training and supervision	No/low incentives for FCHVs Low literacy level of FCHVs	Motivation of FCHVs FCHVs as a part of the existing healthcare system

MHCP, mental healthcare plan; PRIME, PRogramme for Improving Mental Health carE; PHC, primary healthcare; MoH, Ministry of Health; DPHO, district public health office; WHO, World Health Organization, Geneva; mhGAP, Mental Health Gap Action Programme; FCHV, female community health volunteers; CIDT, Community Informant Detection Tool.

The PRIME team provided support for implementation of the packages, including organising training and workshops, managing logistics for training and supervision, and encouraging trained health workers in mental health services delivery. As there was no provision of psychosocial counsellors in the governmental PHC and community healthcare system, PRIME recruited and trained a separate cadre of psychosocial workers to provide psychological interventions in the community as well as in the health facilities where a confidential place was available for psychological intervention. Considering the current lack of mental health supervision in the existing healthcare system, PRIME also took a leading role in the development and implementation of a new supervision system for the trained healthcare workers. Supervision was conducted through monthly/quarterly case conferences led by psychiatrists, face to face and by telephone as needed.

### What were barriers and facilitating factors?

#### Health organisation level

Table 5 presents the barriers and facilitating factors for implementation of each MHCP component. The major barriers for effective implementation of the health-organisation-level intervention packages included mental health not being a government priority, no mental health focal unit/person in the MoH, and lack of basic psychotropic medicines in the free drug list. A memorandum of understanding between PRIME and MoH facilitated the appointment of a senior-level MoH officer to coordinate PRIME activities and procurement of new psychotropic medicines through PRIME. Another key facilitating factor for engagement of senior-level MoH officials was the use of evidence-based intervention packages, particularly the WHO recommended mhGAP intervention guidelines. The supportive role of the district public health office (DPHO) and psychiatrists in the district hospital were other key facilitating factors at this level.

#### Health facility level

The major challenges for implementation of health-facility-level intervention packages included low mental health literacy among PHC workers, heavy workload among PHC workers, frequent transfer of the trained health workers, mental health stigma among service providers, lack of adequate physical facilities, particularly lack of private rooms for consultation, lengthy and complicated drug procurement and distribution process, and lack of a mental health supervision system in primary care. The facilitating factors and strategies adapted to overcome these barriers included: the supportive role of the MoH and DPHO, motivation of PHC workers to learn about mental healthcare, procurement of new psychotropic medicines through PRIME, and initiation of case conferences by psychiatrist for mentoring and clinical supervision of the trained health workers. The feasibility of delivering psychological interventions was another major barrier encountered at this level. This was a barrier for three reasons in particular: first, most of the PHC workers remained busy in out-patient clinics and community outreach activities. Second, PHC workers lack skills to deliver focused psychological interventions. Third, the lack of a private room for providing psychological interventions in the health facilities. To address these barriers, we trained a new cadre of psychosocial counsellors to provide focused psychological support in PHC facilities or in the community setting in case there was no confidential place in the health facilities.

#### Community level

The major barriers for implementation of the community-level intervention packages included limited mental health awareness, low perceived needs for mental healthcare and high level of stigma. The facilitating factors for successful implementation at this level included: involvement of FCHVs, use of CIDT as a strategy to increase demand for services, and mobilisation of a new cadre of psychosocial counsellors to deliver psychological interventions in the community.

## Discussion

The uniqueness of this case study is that it evaluated the impact of a comprehensive district-level MHCP and assessed the barriers and facilitating factors for successful implementation of a MHCP in a real-world setting. The MHCP included four priority disorders, namely psychosis, depression, epilepsy and AUD, recommended by the expert panel,^27^ which was first pilot tested in 2 health facilities, evaluated in 10 facilities, and subsequently scaled up in the entire district (i.e. 34 clinics). The systematic process that we used for development, pilot testing and evaluation of the MHCP was instrumental in getting political buy-in for scaling up of the programme to other districts (*n* = 7).

The area-based approach, which we followed in our study, has also been used in other countries such as Nigeria, Mozambique and Afghanistan, for development and evaluation of mental health services in primary and community care settings. In Nigeria^28^ and Afghanistan^29^ the intervention was tested with priority mental disorders similar to our study, whereas in Mozambique, the pilot programme included epilepsy only.^30^ The results indicate that despite the various contextual, cultural and programmatic challenges, the programme was successful in achieving the intended outcome indicators outlined in the ToC map.^21^ The combination of psychological and mhGAP-based training delivered by mental health specialists was found to be effective for improving mental health knowledge, attitude and the clinical competencies of PHC workers. The barriers and facilitating factors demonstrated by our study are consistent with those reported in Afghanistan, Nigeria and Mozambique.^28–30^

The results show that the number of people receiving primary-care-based mental health services increased significantly after the introduction of the MHCP. On average, patients visited facilities 7.1 times for follow-up care despite a large variation in this number by disorder. About one-third of the patients initiating primary-care-based mental health treatment dropped out after their first visit. These results are consistent with the drop-out rates from mental health services in general medical care reported in Madrid, Spain;^31^ Israel;^32^ and USA.^33^ The possible reasons for a high drop-out rate in our sample could be the availability of a single medicine for each disorder and the frequent transfer of trained health workers. A big drop-out rate for patients with AUD could be explained by health workers losing their faith in treating patients with AUD when many people relapse.^16^ In a nested randomised controlled trial conducted in the PRIME implementation area we demonstrated no added value of community counsellors-delivered psychosocial treatment (i.e. counselling for alcohol problem) over primary health worker-delivered mental healthcare in the treatment of AUD.^25^

Psychotropic medicines were available most of the time in the health facilities, which contrasts with most of the previous studies^34–36^ where supply of psychotropic medicines was one of the major barriers for integration of mental health services in primary care. In Nepal, procurement and distribution of medicines require a lengthy administrative process. However, during the PRIME implementation period, psychotropic medicines were available regularly in most of the health facilities because the PRIME team took a lead role in procurement and distribution of the psychotropic medicines. Now the MoH, provincial government and respective municipalities are sustaining the procurement and distribution of psychotropic medicines. PRIME results support previous evidence that mental health services can be delivered effectively in primary and community healthcare systems in low-resource settings through a task-shifting approach. In our experience, this approach can work effectively only if multiple stakeholders such as mental health specialists, PHC workers and community volunteers are involved in the programme.

### Impact on policy and legislation

The PRIME results and best practices have been used in policies, treatment protocols and guidelines, and training materials by the MoH. First, the PRIME results and best practices have been used in the community mental health care package, which was developed by the Primary Health Care Revitalization Division to facilitate implementation of the National Mental Health Policy (1996).^37^ Second, PRIME results informed the standard treatment protocol that was developed to help PHC workers in detection and treatment of mental health problems.^38^ Third, the essential drugs list has been revised, and six new psychotropic medicines initiated by PRIME have been included. These medicines are now being procured and distributed by the local government (municipalities and village municipalities) and DPHOs. Fourth, the WHO mhGAP Intervention Guide (v2) has been translated and adapted for use in Nepal. The Nepali version of the mhGAP Intervention Guide has added two modules for anxiety disorder and conversion disorders. The MoH has not yet endorsed the adapted Nepali version of the mhGAP Intervention Guide.

Finally, the National Health Training Center, with technical support from Transcultural Psychosocial Organisation Nepal, has developed training manuals and facilitator guides for both PHC and community healthcare workers. These included a training manual for non-prescribers on psychosocial support, a training manual and facilitators’ guides for prescribers, a training manual on advanced psychological interventions (healthy activity program; HAP) and a training manual for FCHVs on the CIDT.

### Limitations

This study has some limitations. First, the evaluation of the MHCP was conducted in ten health facilities in Chitwan. The selection of the district and the area within the district may limit the generalisability of the findings. Second, because of the lack of a baseline on organizational readiness to change, we could not determine whether this affected the MHCP implementation. Finally, although several indicators have helped to explain the success and failure of the MHCP in Nepal, several aspects, which may have contributed to the results, could not be controlled for and tested in the study.

### Policy and practice implications

#### Community level

First, despite the efforts made at the community level to sensitise community members on mental health issues and available services through community awareness and sensitisation programmes, our analysis of the outcomes of the programme published elsewhere showed no significant changes in the treatment coverage and barriers to mental healthcare after implementation of the district MHCP.^6^ A possible explanation could be that the sensitisation and awareness programme primarily aimed to increase mental health literacy and to make people aware about the services available in their community. Previous studies have demonstrated that mental health literacy can change attitudes, but there is no evidence that literacy programmes improve help-seeking intention and behaviour.^39^ There is evidence that help-seeking attitudes and intension can predict behaviour.^40,41^ Therefore, future community interventions should target improving knowledge about mental illness and available services, as well as reducing stigma or negative attitudes towards mental health service utilisation rather than only providing information about mental illness and available services.

Second, it was found that most people receiving mental health treatment from PHCs had a low socioeconomic status. Evidence suggests mental illness and poverty create a vicious cycle that affects the life of people living in poverty and with mental illness throughout the lifespan. Therefore, the programme would have been much more effective for improving the lives of people with mental illness if vocational training for income generation had been included in the in the community-level care package.

Third, only FCHVs were trained on the CIDT, but this approach can be used with other community stakeholders such as mothers’ groups, traditional healers and teachers in the impact in future programmes. This is supported by a study on the accuracy of the CIDT that demonstrated CIDT as an effective tool for detection of people with mental illness in the community.^14^

Fourth, considering the low mental health literacy of non-prescribers and their busy schedule, there is a need for community counsellors to look after psychological intervention in the community. A randomised control trial embedded within the PRIME cohort study demonstrated that a psychological intervention (i.e. HAP) delivered by community-based psychosocial counsellors increased treatment effects for depression compared to those who only received mhGAP-based services in primary care.^16^ In addition, it was also found that because of stigma associated with mental illness and lack of a confidential place in the health facilities for consultation, many patients with mental illness were found to be hesitant to disclose their problems in front of other people. In many health facilities there is no private place for psychological interventions. Dedicated community-based psychosocial counsellors could be a helpful strategy to provide evidence-based psychological interventions in the community, which may also help to minimise the current work burden of PHC workers.

#### Health facility level

First, the 10-day training for prescriber-level health workers was divided into two parts: psychosocial support (5 days) and mhGAP-disorder-specific training (5 days). The psychosocial part of the training was facilitated by a psychologist or an experienced psychosocial counsellor, whereas the mhGAP part was delivered by a psychiatrist. Based on the findings of this study, it would have been more effective if the training had been delivered together by a psychologist and a psychiatrist.

Second, it was not always possible to involve the same psychiatrist in both training and supervision of a trained PHC workers. However, health workers were more comfortable contacting psychiatrists through mobile phone or other means of communication to get support if the same psychiatrist both trained and supervised them. Therefore, we recommend involving the same psychiatrist in both the training and supervision of PHC workers in the impact in future programmes.

Third, the training participants were taken to the district hospital for interaction with actual patients in the initial phase of the mhGAP training. In the later phase, patients were invited to the training venue. Inviting patients to the training venue was much more effective in clarifying various aspects of mental health problems, and the participants also liked this approach better. This approach is recommended for future mhGAP training. In addition, we embedded a study within PRIME in which we trained mental health patients to provide photographic narratives of recovery. Based on a mixed qualitative–quantitative analysis of this proof concept, this approach has potential to improve knowledge, attitudes and clinical competence of PHC workers in mhGAP training.^42^

Fourth, in most of the health facilities, there was no private place for clinical consultation. Because of stigma, patients with mental illness were hesitant to disclose their problems in front of other people; therefore, a separate room should be made available in each health facility for clinical consultation and psychological interventions.

Fifth, despite a very high prevalence of mental health problems among pregnant and postnatal women in Nepal, the data shows that only a small number of them received mental health services from trained health workers. A possible reason could be that pregnant or postnatal women generally consult with non-prescriber-level health workers for pregnancy check-ups, whereas non-prescribers were not trained in diagnosis and management of mental health problems. The non-prescribers should be trained on detection of maternal depression and initiate appropriate psychological interventions. A small pilot study conducted in a few health facilities within PRIME showed that routine screening of perinatal depression and initiation of evidence-based psychological treatment is feasible and effective (results will be published separately). These results are also supported by previous studies where nurses and other lay community health workers delivered psychosocial interventions effectively.^10,43^

Finally, despite the tremendous efforts made by FCHVs to minimise the drop-out rate, about a quarter of the patients initiating primary-care-based mental health services did not come for follow-up. According to FCHVs, patients felt uncomfortable when they made multiple home visits to remind patients about their follow-up care. This could be because of stigma associated with mental illness; therefore, an alternative approach should be developed to minimise the high drop-out rate. One possible strategy is a phone call follow-up by health workers, which could be less stigmatised than FCHVs visiting patients’ house.

#### Health organisation level

First, the PRIME results are based on a model of training all prescribing health workers in a facility, including medical officers (doctors), health assistants and auxiliary health workers. However, the recent treatment protocol endorsed by MoH does not include training auxiliary health workers. Except for a few auxiliary health workers, who were upgraded from other positions, PRIME data shows that auxiliary health workers were the healthcare provider for approximately 60% of all patients in primary care. Despite the benefit of the government taking on more mental health service delivery, the model designed does not match the evidence generated by PRIME. This risks leaving many people without care even in settings where the government mental health model is implemented. This has also raised questions about how to prevent the disconnect between evidence generation and policy making in future.

Second, one of the reasons reported for the high drop-out rate was availability of limited psychotropic medicines (i.e. one medicine for each disorder) in primary care and irregular supply of the medicines. Similarly, frequent transfer of trained health workers was also reported to be another important reason for drop-out. Therefore, regular provision of minimally adequate psychotropic medicines in PHC facilities and regulation of frequent transfer of the trained health workers could help to minimise the high drop-out rate in the impact in future programmes.

Finally, the psychiatrists’ case conference, which was initiated by PRIME for supervision of trained health workers, was found to be effective in building the clinical capacity of the trained healthcare workers and providing specialists care for patients with severe mental health problems. Currently there is no mental health supervision structure for PHC workers in the existing healthcare system; therefore, the ‘psychiatrists’ case conference’ could be an appropriate strategy to fill the current gap in mental health supervision for trained PHC workers.

In conclusion, despite the various contextual challenges, the MHCP resulted in achievement of most of the outcome indicators. The key lessons learned from this study for future integration of mental health services within primary care include the provision of targeted interventions to increase demand for services, and to ensure clinical supervision for health workers, private space for consultations, a separate cadre of psychosocial workers and a regular supply of psychotropic medicines.

## Data Availability

Interested parties may notify the PRIME investigators of their interest in collaboration, including access to the data-set analysed here, through the following website: http://www.prime.uct.ac.za/contact-prime.
